# Draft Genome Sequence of *Megamonas funiformis* Strain Marseille-P3344, Isolated from a Human Fecal Microbiota

**DOI:** 10.1128/genomeA.01459-17

**Published:** 2018-01-11

**Authors:** Mossaab Maaloum, Awa Diop, Sokhna Ndongo, Thi-Tien Nguyen, Frederic Cadoret, Didier Raoult, Pierre-Edouard Fournier

**Affiliations:** a URMITE, Institut Hospitalo-Universitaire Méditerranée-Infection, Aix-Marseille Université, UM63, CNRS 7278, IRD 198, Inserm U1095, Assistance Publique–Hôpitaux de Marseille, Marseille, France; b Faculty of Sciences Ben M’sik, Laboratory of Biology and Health, Hassan II University, Casablanca, Morocco; c Special Infectious Agents Unit, King Fahd Medical Research Center, King Abdulaziz University, Jeddah, Saudi Arabia

## Abstract

In this article, we present the draft genome sequence of *Megamonas funiformis* strain Marseille-P3344, isolated from a human fecal sample. The genome described here is composed of 2,464,704 nucleotides, with 2,230 protein-coding genes and 76 RNA genes.

## GENOME ANNOUNCEMENT

*Megamonas hypermegale* was the first species of the *Megamonas* genus described. The bacterium was isolated from chicken cecum and first described in 1936 as *Bacteroides hypermegas* by Harrison and Hansen (1), and the original name was changed to *Megamonas hypermegale* by Euzéby in 1998 (2). This microorganism is strictly anaerobic and nonmotile. Its optimal growth temperature is 37°C. The species *Megamonas funiformis* was identified in human feces in 2008 in Japan by Sakon et al. (3). Cells from this bacterium are large Gram-negative rods, 5 to 10 μm in size. Some of the cells exhibit a central, subterminal, or terminal swelling of 2- to 4-μm diameter when grown in a broth medium supplemented with glucose.

In August 2016, as part of a microbial culturomics study, we cultivated strain Marseille-P3344 from a fecal sample of a healthy woman. This bacterium exhibited a 99.08% 16S rRNA sequence similarity with *M. funiformis* strain YIT 11815^T^ (=JCM 14723 =DSM 19343), its closest phylogenetic neighbor. Genomic DNA (gDNA) from *M. funiformis* strain Marseille-P3344, isolated from a human fecal specimen, was sequenced using a MiSeq sequencer and the mate pair strategy (Illumina, Inc., San Diego, CA, USA). The gDNA from *M. funiformis* strain Marseille-P3344 was barcoded in order to be mixed with 11 other projects with the Nextera mate pair sample prep kit (Illumina). The gDNA quantification by a Qubit assay with a high-sensitivity kit (Life Technologies, Inc., Carlsbad, CA, USA) was 148.7 ng/µL.

A total of 6.3 Gb was obtained from a 673,000/mm^2^ cluster density with a cluster passing quality control filters of 95.4% (12,453,000 clusters). Within this run, the index representation for *M. funiformis* was 7.99%. The 995,543 mate pair reads were filtered according to the read quality.

The draft genome sequence of *M. funiformis* strain Marseille-P3344 is composed of 7 scaffolds for a total of 2,464,704 nucleotides (nt) and a G+C content of 31.4%. The coding capacity is 2,099,846 nt (85.1% of the total genome). Predicted genes include 2,230 protein-coding genes, of which 1,701 are assigned to clusters of orthologous groups and 76 (3.29%) are RNA genes (17 rRNAs and 59 tRNAs). A total of 228 genes (10.2%) have peptide signals, and 481 (21.5%) have transmembrane helices. In addition, 46 virulence genes are predicted, including 30 genes associated with antibiotic resistance, including 3 beta-lactamases. No toxin/antitoxin module or bacteriocin-associated gene could be found.

The genomes of *M. funiformis* strains Marseille-P3344 and YIT 11815^T^ (=JCM 14723 =DSM 19343) were compared using GGDC and OrthoANI softwares (4, 5). Digital DNA-DNA hybridization and OrthoANI values of 84.1% ± 2.6 (>70%) and 98.18% (>95.96%), respectively, were obtained, thus confirming that these strains belong to the same species.

### Accession number(s).

The 16S rRNA and whole-genome sequences reported here have been deposited in GenBank under accession numbers LT628480 and FQRY00000000, respectively.
